# Prospective evaluation of combination oral S-1 and intravenous cisplatin in cancer patients with severe hepatic dysfunction – a feasibility, safety, and pharmacokinetic study

**DOI:** 10.3389/fonc.2026.1948986

**Published:** 2026-10-16

**Authors:** Yong Xiang Gwee, Andrea Li Ann Wong, Boon Cher Goh, Ross Andrew Soo, Wei Peng Yong, David Shao Peng Tan, Cheng Ean Chee, Siew Eng Lim, Yvonne Ang, Yiqing Huang, Wan Qin Chong, Joline Lim, Gloria Chan, Natalie Ngoi, Samuel Ow, Rachel Wong, Kenneth Sooi, Joan Choo, Soo Chin Lee

**Affiliations:** Department of Haematology-Oncology, National University Cancer Institute, Singapore, Singapore

**Keywords:** cisplatin, feasibility study, hepatic dysfunction (HD), pharmacokinetic, S-1 (tegafur/gimeracil/oteracil)

## Abstract

**Introduction:**

Patients with advanced solid tumors and severe hepatic dysfunction have limited systemic therapy options due to impaired drug metabolism and increased toxicity risks. This population is often excluded from clinical trials, leaving a critical treatment gap. This study evaluates the feasibility, safety, pharmacokinetics and efficacy of S-1, an oral prodrug of 5-fluorouracil (5-FU), plus cisplatin in these patients.

**Methods:**

Between September 2016 and November 2023, 61 patients were enrolled into four cohorts based on hepatic function per NCI-ODWG criteria: normal (Cohort 1), mild (Cohort 2), moderate (Cohort 3), and severe dysfunction (Cohort 4). Primary endpoints were safety and 5-fluorouracil pharmacokinetics; secondary endpoints included objective response rate (ORR) and 3-month clinical benefit rate (3M-CBR).

**Results:**

On Cycle 1 day 15, patients with severe hepatic dysfunction (Cohort 4) had lower 5-FU C_max_ and longer T_max_ than those with normal hepatic function (Cohort 1) (p<0.05), though 5-FU AUC remained comparable across cohorts. Safety in Cohort 4 were similar to those in Cohorts 1–3. No grade 5 treatment-related adverse events occurred. In Cohort 4, 22.2% achieved a partial response, 40.7% derived clinical benefit, and 22.2% maintained progression-free survival ≥6 months, providing preliminary evidence of clinical activity in this population.

**Conclusion:**

Patients with severe hepatic dysfunction had similar 5-FU AUC and toxicities to those with less severe hepatic dysfunction, and preliminary evidence of clinical activity was observed with cisplatin + S-1. These findings provide prospective preliminary evidence that this combination may be feasible in selected patients with severe hepatic dysfunction, a population frequently excluded from clinical trials. Larger studies are needed to further define its safety, pharmacokinetics, and clinical activity in this population.

(Clinical Trial Registration: NCT03519074. Retrospectively registered 08 May 2018).

## Introduction

Hepatic dysfunction is a common co-morbidity in patients with advanced cancer, particularly in those with extensive liver metastases. Many systemic therapies rely on hepatic metabolism, limiting treatment options in patients with severe hepatic dysfunction and increasing the risk of toxicity. These patients are frequently excluded from clinical trials, leaving a major evidence gap in clinical decision-making.

Identifying safe and effective systemic therapies in this population is therefore critical. S-1, an oral fluorinated pyrimidine agent, is one such candidate due to its unique pharmacological properties. S-1 contains three main ingredients: tegafur, gimeracil (CDHP), and potassium oxonate (Oxo). Tegafur, an oral pro-drug of 5-fluorouracil (5-FU), is converted to its active metabolite in tumor tissues. Gimeracil maintains high concentrations of 5-FU systemically by inhibiting dihydropyrimidine dehydrogenase (DPD), a major catabolic enzyme of 5-FU found in high concentrations in the liver. Oxo, the third ingredient in oral S-1, inhibits orotate phosphoribosytransferase (ORPT) in the gastrointestinal tract to reduce gastrointestinal toxicities, without affecting anti-tumor activity (1). S-1 has demonstrated activity across multiple solid tumors, including gastric, colorectal, breast, non-small cell lung, pancreatic, and head and neck cancers (2–6).

Despite its broad-spectrum activity, S-1 has not been formally studied in cancer patients with hepatic dysfunction, leaving unclear guidance regarding appropriate dosing and safety in this population. While no specific dosage adjustments are recommended, fluoropyrimidines are generally used cautiously in severe hepatic dysfunction (7). S-1 is mainly metabolized by CYP2A6, a major cytochrome P450 isoenzyme in human liver microsomes. In addition, urinary excretion of gimeracil is markedly decreased in severe renal impairment. Thus, caution has to be exercised when administering S-1 in the presence of severe renal and/or hepatic dysfunction (8). Cisplatin is an active chemotherapeutic agent with broad spectrum activity that can safely be administered in severe hepatic dysfunction. The combination of cisplatin and S-1 has demonstrated efficacy in patients with preserved organ function (9–12).

We conducted this prospective phase II study to evaluate the safety, pharmacokinetics, and efficacy of S-1 plus cisplatin in patients with varying degrees of hepatic dysfunction, including those with severe dysfunction. These findings aim to inform clinical decision-making in this underserved population.

## Methods

### Patients

Eligible patients were 18 years or older with histologically or cytologically confirmed solid tumors that were refractory to standard therapy or had no available treatment options. Additional requirements include measurable disease using Response Evaluation Criteria in Solid Tumors (RECIST) criteria version 1.1 (13), an Eastern Cooperative Oncology Group performance-status score of 0 or 1 (14), an estimated life expectancy of at least 12 weeks, and adequate bone marrow (absolute neutrophil count ≥ 1.5 x 10^9^/L and platelets ≥ 100 x 10^9^/L) and renal function (calculated creatinine clearance >60ml/minute).

Exclusion criteria included impairment of gastrointestinal (GI) function or GI disease that may significantly alter the absorption of S-1 (e.g., ulcerative diseases, uncontrolled nausea, vomiting, diarrhea, malabsorption syndrome, or small bowel resection), concurrent administration of any other tumor therapy (including cytotoxic chemotherapy, hormonal therapy, and immunotherapy), treatment with a small molecule targeted agent ≤2 weeks prior to starting study treatment, treatment within the last 30 days with any investigational drug, radiotherapy ≤4 weeks prior to starting study treatment, have not recovered from radiotherapy-related toxicities (limited field palliative radiotherapy ≤2 weeks prior to starting study treatment was allowed), major surgery within 28 days of study drug administration, history or presence of serious uncontrolled ventricular arrhythmias, a history of human immunodeficiency virus infection or symptomatic brain metastasis. Patients with active infection, including active hepatitis B or C infection or cholangitis, were also excluded. Patients with malignant biliary obstruction were eligible only after biliary drainage has been performed, where clinically indicated. In such patients, liver function tests were repeated following biliary drainage after an appropriate interval as deemed by the study investigator(s), to allow for potential biochemical recovery, and hepatic dysfunction cohort assignment was subsequently based on the post-drainage liver function according to the NCI-ODWG criteria. This approach was intended to minimize the influence of potentially reversible biliary obstruction on the classification of baseline hepatic dysfunction.

The study is registered at ClinicalTrials.gov (NCT03519074; retrospectively registered 08 May 2018), and the study protocol was approved by the Domain Specific Review Board, National Healthcare Group, Singapore (2016/00691). All research was performed in accordance with relevant guidelines/regulations and was conducted in full compliance with the Declaration of Helsinki. All patients provided written, informed consent prior to study entry.

### Trial design and treatment

This was a single-arm, open-label phase II study. Patients were stratified into four cohorts based on hepatic function using National Cancer Institute Organ Dysfunction Working Group (NCI-ODWG) criteria (**Supplementary Table 1**), which provides standardized classifications of hepatic dysfunction severity.

A 3 + 3 design was used to assess safety. Cohorts were expanded after initial safety evaluation, with larger planned enrollment for patients with severe hepatic dysfunction to address the limited data in this population. We aimed to enroll up to 12 patients in cohorts 1 (normal hepatic function) and 2 (mild hepatic dysfunction), for a total of at least 10 patients with pharmacokinetics (PK) data and evaluable for toxicity in each group. Up to 25 patients were to be enrolled into cohort 3 (moderate hepatic dysfunction) and 35 patients into cohort 4 (severe hepatic dysfunction), to achieve at least 15 patients with PK data and evaluable for toxicity in each group. Cohort 4 had a larger planned sample size due to anticipated higher attrition rates, to ensure adequate evaluation of safety, efficacy, and pharmacokinetics.

Patients received intravenous cisplatin 60mg/m^2^ on day 1 with oral S-1 30mg/m^2^ twice daily on days 1–14 of each 21-day cycle, until disease progression or intolerable toxicities. After six cycles of cisplatin plus S-1, in the absence of progressive disease, cisplatin could be discontinued while continuing oral S-1 30mg/m^2^ twice daily on days 1–14 of each cycle, every three weeks.

### Definition of dose limiting toxicities

DLTs were assessed during cycle 1 and graded based on Common Terminology Criteria for Adverse Events (CTCAE) version 4.03. DLTs included severe hematologic toxicity, grade ≥3 non-hematologic toxicity despite optimal supportive care, clinically significant renal impairment, or treatment-related worsening of hepatic function beyond predefined thresholds adjusted for baseline hepatic function. Detailed cohort-specific definitions are provided in the Study Protocol. Decline in hepatic function deemed by study investigators to be primarily related to rapid disease progression, resulting in inability to complete first cycle of study treatment, would not constitute DLT.

### Patient evaluation

At baseline and before each treatment cycle, patients underwent history-taking, physical examination, performance status assessment, and laboratory evaluations, including full blood count, liver and renal function tests, and electrolytes. Pregnancy testing was performed where applicable. Safety assessments were performed weekly during the first treatment cycle and once per subsequent cycle, with adverse events graded per CTCAE v4.03. All patients receiving at least one dose of study drug were included in safety analyses. Patients who withdrew before treatment or before disease assessment were replaced. Tumor response was evaluated radiographically every two cycles (approximately every 6 weeks) for the first 6 months, and every 12 weeks thereafter, at the investigator’s discretion. Patients were evaluable for treatment response if they completed at least two cycles and had post-treatment imaging or if progression occurred before two full cycles. Patients lost to follow-up before response evaluation were replaced for efficacy endpoints.

### Study objectives and end points

The primary objectives of this study were to evaluate the safety and determine the pharmacokinetics of S-1 in patients with advanced solid tumors with varying degrees of hepatic dysfunction. Secondary objectives were to evaluate objective response rate (ORR), 3-month clinical benefit rate (3M-CBR) and progression-free survival (PFS) of patients treated with this combination therapy. ORR was defined as the percentage of patients who have at least one confirmed response of Complete Response (CR) or Partial Response (PR). 3M-CBR was defined as the percentage of patients who achieved at least one confirmed response of CR, PR or Stable Disease (SD) lasting for at least 3 months. PFS was defined as the interval from study enrollment to progression of disease or death from any cause, whichever occurred earlier.

### Pharmacokinetic methods

5-FU pharmacokinetic sampling was performed on all patients at the following time points: pre-treatment, after 7 days and 14 days dosing of S-1 during cycle 1 at 0, 30 minutes, 60 minutes, 2 hours, 4 hours and 6 hours after dosing. Blood samples were collected via venepuncture or indwelling venous lines and processed for analysis. PK assays were performed at the Cancer Science Institute, National University of Singapore. Individual parameter estimates were determined by standard compartmental and non-compartmental methods based on WINNONLIN software. Plasma concentrations of 5-FU and its metabolites were measured by liquid chromatography with tandem mass spectrometry (LC/MS/MS).

### Genotyping and pharmacogenomic analysis

To explore genetic contributions to treatment variability, genomic DNA was extracted from pre-treatment whole blood samples followed by genome-wide genotyping performed using the Infinium Global Screening Array-24 v3.0 BeadChip (Illumina, San Diego, CA). Sample processing followed the Infinium HTS protocol: DNA amplification, fragmentation, hybridization to the array, single-base extension, staining, and imaging on the Illumina iScan system. Five single nucleotide polymorphisms (SNPs) in the CYP2A6 gene were selected *a priori* based on known or suspected functional relevance: rs28399433, rs8192720, rs28399440, rs5031016, and rs28399468 (15, 16). Associations between genotypes and clinical outcomes (ORR, 3M-CBR, and ≥grade 3 toxicity) were evaluated using Fisher’s exact test. Statistical analyses were conducted using IBM^®^ SPSS^®^ Statistics Version 29.0.2.0, with two-sided p-values <0.05 considered statistically significant.

### Statistical analysis

Pairwise comparisons of pharmacokinetic variables between hepatic function cohorts (Cohorts 1 vs. 2, 1 vs. 3, and 1 vs. 4) were conducted using independent samples t-tests to evaluate the impact of hepatic dysfunction on drug metabolism. Associations between 5-FU exposure, toxicity, and clinical outcomes were explored using logistic regression. To identify baseline factors predictive of clinical benefit in Cohort 4, univariate logistic regression was performed. Factors with significant associations were further assessed using multivariate logistic regression to adjust for potential confounders. All analyses were two-tailed with a significance level of *p* < 0.05.

## Results

### Patients and treatment

Between September 2016 and November 2023, 61 patients were enrolled across four cohorts. Baseline characteristics are summarized in **Table 1**. The median age was 55 years, most patients had ECOG performance status 1, and breast cancer was the most common tumor type. The median baseline total bilirubin in Cohort 4 was 3.4 x ULN (range 0.3-8.2). Median follow-up duration was 3.7 months (range 0.1 – 95.4) across all patients, and 5.1 months (range 2.3 – 95.4), 4.3 months (range 2.8 – 18.6), 3.4 months (range 0.5 – 20.0) and 3.7 months (range 0.1 - 30.4) across Cohorts 1 to 4 respectively.

**Table 1:** Characteristics of Patients.

	Cohort 1	Cohort 2	Cohort 3	Cohort 4	Total
No. of patients	11	10	13	27	61
Median age (range) – yr	51 (42 – 59)	46 (27 – 62)	55 (48 – 68)	57 (23 – 75)	55 (23 – 75)
Sex – No. (%)					
Male	2 (18.2)	1 (10)	4 (30.8)	9 (33.3)	16 (26.2)
Female	9 (81.8)	9 (90)	9 (69.2)	18 (66.7)	45 (73.8)
Ethnicity – no. (%)					
Chinese	9 (81.8)	7 (70)	11 (84.6)	14 (51.9)	41 (67.2)
Malay	2 (18.2)	0	1 (7.7)	7 (25.9)	10 (16.4)
Indian	0	0	0	3 (11.1)	3 (4.9)
Others	0	3 (30)	1 (7.7)	3 (11.1)	7 (11.5)
ECOG – No. (%)					
0	3 (27.3)	6 (60)	1 (7.7)	1 (3.7)	11 (18.0)
1	7 (63.6)	4 (40)	10 (76.9)	15 (55.6)	36 (59.0)
2	0	0	0	0	0
3	0	0	0	1 (3.7)	1 (1.6)
Missing	1 (9.1)	0	2 (15.4)	10 (37.0)	13 (21.3)
Histology – No. (%)					
Breast	7 (63.6)	8 (80)	5 (38.5)	15 (55.6)	35 (57.4)
Biliary Tract	0	0	1 (7.7)	4 (14.8)	5 (8.2)
Colorectal	0	0	3 (23.1)	2 (7.4)	5 (8.2)
Pancreas	0	0	2 (15.4)	2 (7.4)	4 (6.6)
Head & Neck	1 (9.1)	1 (10)	1 (7.7)	1 (3.7)	4 (6.6)
Lung	1 (9.1)	0	1 (7.7)	1 (3.7)	3 (4.9)
HCC	0	0	0	2 (7.4)	2 (3.3)
Esophagogastric	2 (18.2)	0	0	0	2 (3.3)
Ovarian	0	1 (10)	0	0	1 (1.6)
Prior lines of treatment – Median (Range)	3 (0 – 8)^a,b^	5 (0 – 9)^a^	4 (2 – 12)	2 (0 – 9)	3 (0 – 12)
Baseline Albumin – Median (range) (g/L)	36 (31 – 44)	39 (30 – 44)	36 (25 – 41)	32 (22 – 38)	35 (22 – 44)
Baseline BMI – Median (range) (kg/m^2^)	23.8 (14.8 – 38.7)	20.7 (14.1 – 30.6)	22.4 (16.1 – 27.1)	22.9 (15.0 – 35.2)	22.4 (14.1 – 38.7)
Baseline total bilirubin (x ULN) – Median (Range)	0.3 (0.2 – 0.5)	0.3 (0.3 – 1.0)	2.1 (1.6 – 2.9)	3.4 (0.3 – 8.2)	2.0 (0.2 – 8.2)
Baseline AST (x ULN) – Median (Range)	0.5 (0.2 – 0.9)	1.4 (1.0 – 4.0)	2.9 (1.2 – 4.3)	5.9 (1.4 – 21.6)^c^	2.9 (0.2 – 21.6)
Baseline ALT (x ULN) – Median (Range)	0.2 (0.1 – 0.4)	0.6 (0.3 – 3.8)	1.3 (0.5 – 4.4)	1.8 (0.5 – 9.4)	1.4 (0.1 – 9.4)

^a^ In Cohorts 1 and 2, there were four patients in total who were treatment-naïve prior to trial enrolment. These included one patient with relapsed oesophageal squamous cancer diagnosed in 2017 (Cohort 1), one patient with relapsed metastatic oral squamous cell carcinoma diagnosed in 2017 (Cohort 1) and one patient with metastatic nasopharyngeal cancer diagnosed in 2018 (Cohort 2) for whom the treating physician deemed the study treatment combination of cisplatin and S-1 as suitable for front-line systemic chemotherapy treatment at the time of diagnosis. There was also one patient with HER2-positive breast cancer with liver and bone metastases (Cohort 2) who declined front-line trastuzumab-based combination therapy in 2017 at point of diagnosis due to cost considerations, and was hence offered enrolment onto this clinical trial.

^b^ In Cohort 1, there were two patients with only one prior line of treatment prior to trial enrolment. These included one patient with triple-negative breast cancer who progressed on front-line chemotherapy in 2016 (immunotherapy and antibody-drug conjugate therapies were not standard treatments in 2016, when the patient was enrolled) and one patient with *PIK3CA-*mutant hormone receptor-positive breast cancer for whom, following progression on a clinical trial of paclitaxel combined with an AKT inhibitor, the treating team considered combination chemotherapy to be appropriate subsequent therapy, and the patient was enrolled onto the present study.

^c^ Deviation from study protocol occurred for 1 patient in Cohort 4. Patient #31 with baseline AST >20x ULN was enrolled onto the study.

AST, Aspartate transaminase; ALT, Alanine transaminase; ULN, Upper Limit of Normal.

To provide a complementary assessment of hepatic function, ALBI grade was determined according to baseline serum albumin and bilirubin concentrations for all patients. The distribution of ALBI grades across the four NCI-ODWG hepatic dysfunction cohorts is shown in **Supplementary Table 2**. Overall, ALBI grade increased with increasing severity of NCI-ODWG hepatic dysfunction. No patients in Cohorts 1 or 2 had ALBI grade 3, whereas ALBI grade 3 was observed in 1 of 13 patients (8%) in Cohort 3 and 13 of 27 patients (48%) in Cohort 4. All patients in Cohorts 3 and 4 were classified as ALBI grade 2 or 3. These findings provide descriptive evidence of broad concordance between the NCI-ODWG classification used for cohort assignment and ALBI grading. However, the two classification systems assess hepatic function using different parameters and thresholds, and the small sample size precludes formal assessment of concordance or validation between the two approaches.

### Safety

DLTs occurred in two patients (3.3%)—one in Cohort 2 (10%) and one in Cohort 3 (7.7%). The patient in Cohort 2 was unable to complete cycle 1 treatment due to G3 fatigue. The patient in Cohort 3 experienced G3 vomiting attributed to cisplatin. Beyond DLTs, 11 additional patients (2 in Cohort 3 [15.4%] and 9 in Cohort 4 [33.3%]) discontinued treatment in cycle 1 due to rapid disease progression.

Treatment-related adverse events (TRAEs) are summarized in **Table 2**. TRAEs occurred in 83.6% of patients, with 47.5% experiencing grade 3–4 events. Common toxicities included hematologic effects, fatigue, gastrointestinal symptoms, and electrolyte abnormalities. No treatment-related deaths occurred.

**Table 2:** Treatment-related adverse events (TRAEs) during chemotherapy.

	Cohort 1		Cohort 2		Cohort 3		Cohort 4	
Number of patients	11		10		13		27	
Median cycles of chemotherapy received (range)	3 (2 – 11)		4 (2 – 6)		2 (1 – 22)		2 (1 – 28)	
TRAEs – no. (%)	All Grade	G3/4	All Grade	G3/4	All Grade	G3/4	All Grade	G3/4
Any TRAEs	11 (100)	8 (73)	9 (90)	3 (30)	10 (77)	6 (46)	21 (78)	12 (44)
Haematologic								
Neutropenia	5 (45)	**4 (36)**	2 (20)	**23 (20)**	2 (15)	**2 (15)**	9 (33)	**6 (22)**
Anemia	1 (9)	1 (9)	2 (20)	**2 (20)**	3 (23)	**2 (15)**	4 (15)	**3 (11)**
Thrombocytopenia	0	0	1 (10)	0	4 (31)	**2 (15)**	4 (15)	**3 (11)**
Non-hematologic								
Fatigue	7 (64)	**2 (18)**	8 (80)	**1 (10)**	7 (54)	**2 (15)**	12 (44)	2 (7)
Anorexia	4 (36)	0	0	0	2 (15)	0	1 (4)	0
Nausea	4 (36)	0	6 (60)	0	3 (25)	1 (8)	3 (11)	0
Vomiting	2 (18)	0	1 (10)	0	1 (8)	1 (8)	2 (7)	0
Constipation	1 (9)	0	3 (30)	0	1 (8)		2 (7)	0
Diarrhoea	1 (9)	0	1 (10)	0	0	0	4 (15)	1 (4)
Hyponatremia	2 (18)	1 (9)	1 (10)	**1 (10)**	0	0	0	0
Hypokalemia	0	0		0	0	0	4 (15)	2 (7)
Hypomagnesemia	0	0	1 (10)	0	0	0	1 (4)	0
Hypercalcemia	1 (9)	0	0	0	0	0	0	0
Dysgeusia	0	0	0	0	1 (8)	0	3 (11)	0
Dehydration	1 (9)	1 (9)	0	0	0	0	1 (4)	1 (4)
Loss of weight	1 (9)	0	0	0	0	0	0	0
Oral mucositis	0	0	0	0	0	0	3 (11)	1 (4)
Peripheral neuropathy	3 (27)	1 (9)	0	0	1 (8)	0	2 (7)	0
Myalgia	0	0	0	0	0	0	1 (4)	0
Weakness	1 (9)	1 (9)	0	0	0	0	0	0
Fever (non-neutropenic)	1 (9)	0	0	0	0	0	0	0
GORD	0	0	1 (9)	0	0	0	0	0
Hyperpigmentation	0	0	2 (18)	0	0	0	0	0
Oedema	0	0	0	0	1 (8)	0	2 (7)	0
Epistaxis	0	0	1 (9)	0	0	0	0	0
Abdominal pain	0	0	1 (9)	0	0	0	0	0
Rash	2 (18)	0	0	0	0	0	0	0
Hearing impairment	1 (9)	0	0	0	0	0	1 (4)	0
Tinnitus	0	0	0	0	1 (8)	0	0	0
Pruritus	0	0	0	0	1 (8)	0	0	0
Dry skin	0	0	0	0	2 (17)	0	0	0
Raised troponin	0	0	0	0	0	0	1 (4)	0
Hiccups	0	0	0	0	0	0	1 (4)	0
AKI	0	0	0	0	0	0	1 (4)	1 (4)
Dry eyes	0	0	0	0	1 (8)	0	0	0
Giddiness	0	0	0	0	1 (8)	0	0	0
Tachycardia	0	0	0	0	0	0	1 (4)	1 (4)
Raised lactate	0	0	0	0	0	0	1 (4)	1 (4)

≥G3 AEs with frequency ≥10% highlighted in **bold.**

### Pharmacokinetics

Pharmacokinetic variables of 5-fluorouracil across cohorts on days 8 and 15 of S-1 dosing are summarized in **Tables 3A** and **3B**. **Figures 1A, B** illustrate the mean plasma concentration-time profiles. Pharmacokinetic data were available for 41 patients at day 8 and 37 patients at day 15. Data from two Cohort 4 patients were excluded due to treatment non-compliance.

**Table 3A:** 5-Fluorouracil Pharmacokinetic Parameters – at Cycle 1 Day 8.

	No. of patients	C_max_ (ng/mL) (Mean ± SD)	*p*-value (vs. Cohort 1)	T_max_ (hours) (Mean ± SD)	*p*-value (vs. Cohort 1)	AUC_0-6h_ (h*ng/mL) (Mean ± SD)	*p*-value (vs. Cohort 1)
Cohort 1	10	194.5 ± 60.2	–	1.8 ± 0.9	–	679.0 ± 121.4	–
Cohort 2	8	191.0 ± 87.5	0.921	2.5 ± 1.7	0.278	653.9 ± 249.1	0.782
Cohort 3	8	172.3 ± 60.1	0.449	2.8 ± 1.0	0.056	689.1 ± 294.3	0.922
Cohort 4	15	158.1 ± 70.4	0.194	2.7 ± 1.2	0.062	616.2 ± 243.0	0.459

**Table 3B:** 5-Fluorouracil Pharmacokinetic Parameters – at Cycle 1 Day 15.

	No. of patients	C_max_ (ng/mL) (Mean ± SD)	*p*-value (vs. Cohort 1)	T_max_ (hours) (Mean ± SD)	*p*-value (vs. Cohort 1)	AUC_0-6h_ (h*ng/mL) (Mean ± SD)	*p*-value (vs. Cohort 1)
Cohort 1	10	199.9 ± 58.4	–	1.9 ± 0.9	–	738.4 ± 247.7	–
Cohort 2	9	173.8 ± 84.5	0.440	1.9 ± 0.9	0.979	668.0 ± 382.8	0.636
Cohort 3	8	184.3 ± 81.8	0.643	1.7 ± 0.6	0.566	704.4 ± 269.7	0.784
Cohort 4	10	135.8 ± 52.3	**0.018**	3.1 ± 1.5	**0.045**	545.4 ± 227.4	0.086

**Figure 1:**
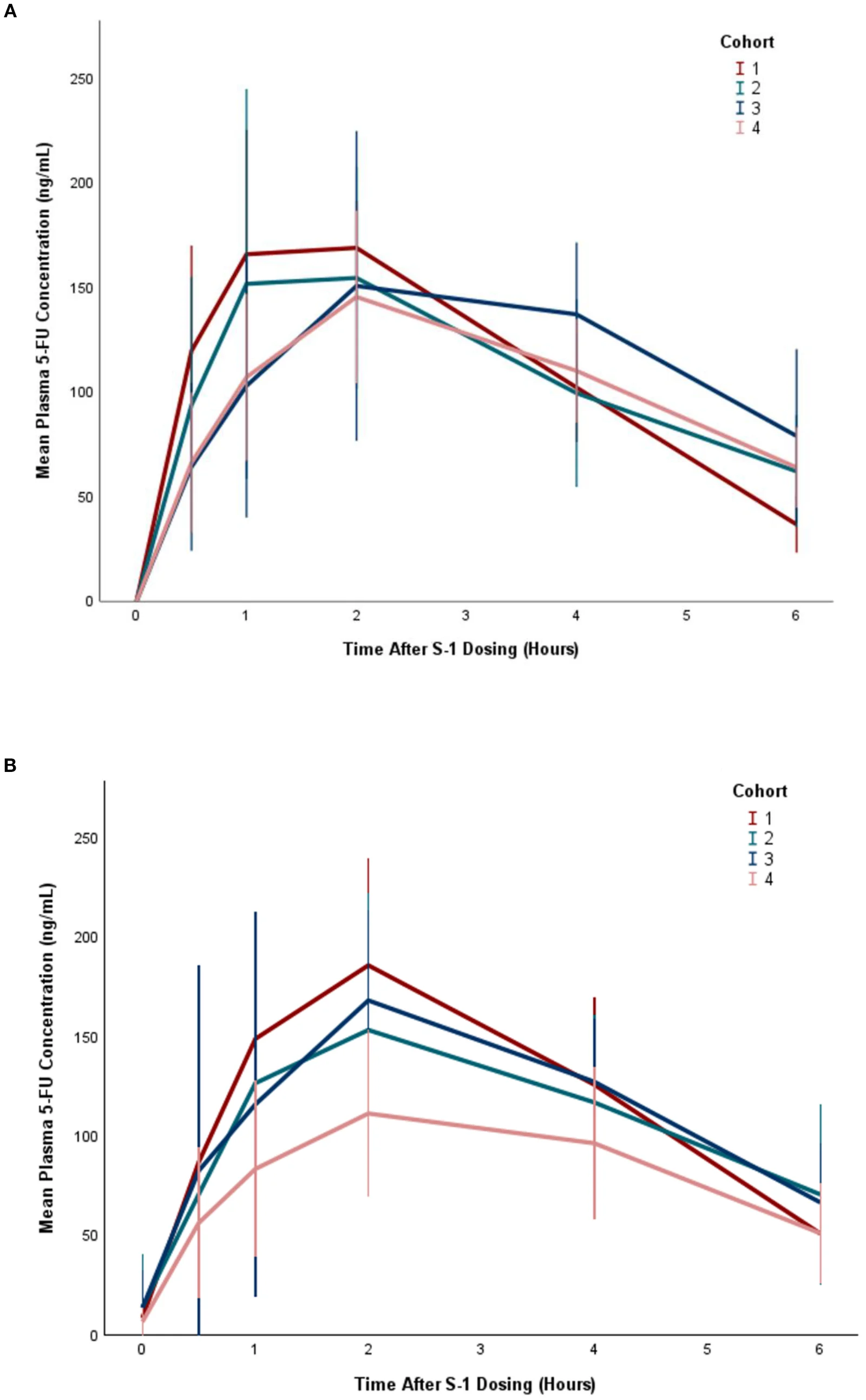
**(A)** 5-Fluorouracil (5-FU) Plasma Concentration-Time Plot at Cycle 1 Day 8. **(B)** 5-Fluorouracil (5-FU) Plasma Concentration-Time Plot at Cycle 1 Day 15.

No significant pharmacokinetic differences were observed between Cohorts 1 and 2 or Cohorts 1 and 3 at days 8 and 15. On the other hand, compared to Cohort 1, Cohort 4 patients exhibited similar 5-FU pharmacokinetics at day 8, a trend towards lower AUC at day 15 (*p* = 0.086), but significantly lower 5-FU C_max_ and longer T_max_ on day 15 (*p* < 0.05), suggesting altered S-1 metabolism in severe hepatic dysfunction.

An exploratory analysis was performed to assess correlations between pharmacokinetic parameters of 5-FU and clinical benefit from treatment (**Supplementary Table 3**). Univariate analysis showed that shorter T_max_ and higher C_max_ on Day 8 were associated with clinical benefit, but these were not significant in multivariate analysis. No association was observed between Day 15 C_max_ or T_max_ and clinical benefit.

Similarly, exploratory analysis examining correlations between pharmacokinetic variables of 5-fluorouracil and treatment-related toxicities is summarized in **Supplementary Table 4**. While shorter T_max_ and higher AUC on Day 8 showed association with severe (≥ Grade 3) treatment-related toxicity in univariate analysis, these associations were not significant in multivariate analysis. There was no association between Day 15 C_max_ or T_max_ with severe treatment-related toxicities.

Finally, univariate analysis of Cohort 4 patients (**Supplementary Table 5**) indicated that lower baseline total bilirubin, higher ALT levels, higher 5-FU C_max_, and AUC on Day 8 were associated with treatment response. However, none remained significant in the multivariate model.

### Efficacy

ORR ranged from 0–22.2%, and 3M-CBR ranged from 15.4–60% across cohorts (**Table 4**). Median PFS ranged from 1.83 to 3.00 months (**Figure 2A**), and median overall survival (OS) ranged from 2.83 to 6.13 months (**Figure 2B**). However, these outcomes were secondary endpoints and were not designed to establish differences in prognosis between cohorts. Baseline characteristics differed between cohorts (**Table 1**), including tumor type and prior treatment exposure. Notably, the median number of prior treatment lines was 4 (range, 2–12) in Cohort 3 compared with 2 (range, 0–9) in Cohort 4. Given these differences and the small cohort sizes, survival outcomes should be interpreted descriptively.

**Table 4:** Response Outcomes to Cisplatin and S-1.

	Cohort 1	Cohort 2	Cohort 3	Cohort 4
No. of patients	11	10	13	27
Median Progression Free Survival – months (95% CI)	3.00 (2.10 – 3.90)	3.00 (2.33 – 3.67)	1.83 (1.17 – 2.49)	2.80 (1.55 – 4.05)
Median Overall Survival – months (95% CI)	5.77 (1.45 – 10.08)	6.13 (5.06 – 7.21)	2.83 (2.61 – 3.05)	3.37 (1.78 – 4.95)
Objective response				
No. of patients	2	2	0	6
% (95% CI)	18.2 (5.1 – 47.7)	20.0 (5.7 – 51.0)	0.0 (0 – 22.8)	22.2 (10.6 – 40.8)
3-Month Clinical Benefit – no. (%)				
No. of patients	6	6	2	11
% (95% CI)	54.5 (28.0 – 78.7)	60.0 (31.3 – 83.2)	15.4 (4.3 – 42.2)	40.7 (24.5 – 59.3)
Best overall response – no. (%)				
Partial response	2 (18.2)	2 (20.0)	0	6 (22.2)
Stable disease	6 (54.5)	6 (60.0)	5 (38.5)	8 (29.6)
Progressive disease	2 (18.2)	2 (2.00)	7 (53.8)	9 (33.3)
No assessment	1 (9.1)	0	0	0
Not evaluable	0	0	1 (7.7)^a^	4 (14.8)^b^
Patients who received subsequent treatment post-study – no. (%)	4 (36.4)	5 (50.0)	1 (7.7)	6 (18.5)

^a^ One patient in Cohort 3 withdrew from study after cycle one of treatment.

^b^ Of these four patients in Cohort 4, two patients withdrew from study after cycle one of treatment and two patients were lost to follow-up after cycle one.

**Figure 2:**
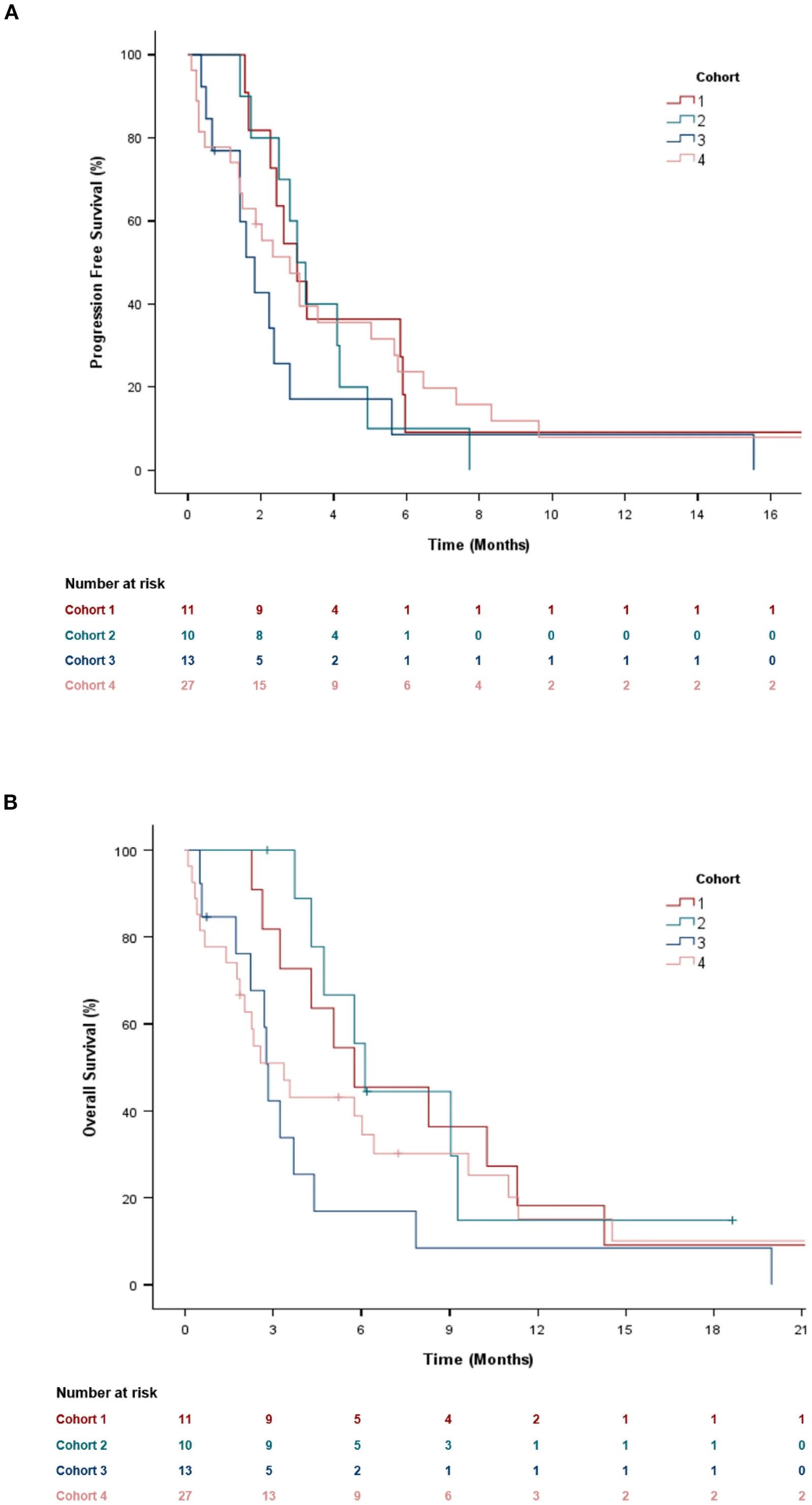
**(A)** Kaplan-Meier Estimates of Progression-Free Survival (PFS), according to Cohorts of Varying Hepatic Dysfunction **(B)** Kaplan-Meier Estimates of Overall Survival (OS), according to Cohorts of Varying Hepatic Dysfunction.

Despite severe hepatic dysfunction, promising anti-tumour activity was observed in Cohort 4 patients. ORR was 22.2%, 3M-CBR was 40.7% and 22.2% (6/27) had PFS ≥6 months (**Figure 2**). Among the six Cohort 4 patients with partial response, five had breast cancer and one had nasopharyngeal carcinoma. Notably, one patient had baseline total bilirubin 4.13x ULN, while the other five had baseline AST and/or ALT >5x ULN. Among breast cancer patients in Cohort 4 (n=15), ORR was 33.3% and 3M-CBR was 46.7%, compared with ORR 8.3% and 3M-CBR 33.3% in non-breast cancers (n=12), without a statistically significant difference (*p* = 0.696).

**Supplementary Figure 1** illustrates the treatment response of patients in Cohort 4 who were evaluable for response, while **Supplementary Figures 2A–C** depict hepatic function trends among those with 3-month clinical benefit. These plots demonstrate the changes in hepatic biochemical parameters during treatment and provide a descriptive assessment of hepatic function over the early treatment period. Notably:

• 29.4% (5/17) of patients with baseline total bilirubin >3× ULN derived 3-month clinical benefit, including one partial response and four sustained stable disease.
• 40.0% (6/15) of patients with baseline AST >5× ULN achieved clinical benefit, including five partial responses.
• Among four patients with ALT >5× ULN (all of whom had AST >5× ULN), 75.0% achieved partial response.
• Individual hepatic biochemical trajectories demonstrated variable changes in bilirubin, AST and ALT during the first two treatment cycles, with reductions in these parameters observed in several patients who achieved 3-month clinical benefit.

### CYP2A6 polymorphisms and clinical associations

Of 61 patients, 58 had suitable blood samples for genotyping. The functional impact of the five SNPs of interest included decreased mRNA expression, reduced or inactive enzyme activity (**Supplementary Table 6**).

The most prevalent variant was rs28399433 (A>C), observed in 21 patients (36.2%; all heterozygous), followed by rs8192720 (G>A) in 11 patients (19.0%; 9 heterozygous, 2 homozygous) and rs5031016 (A>G) in 7 patients (12.1%; all heterozygous). Variants rs28399440 and rs28399468 were rare, with allele frequencies of 1.7% and 3.4% (all heterozygous), respectively. All SNPs were in Hardy-Weinberg equilibrium (HWE), with *p*-values ranging from ~1.0 to 0.055, suggesting no significant deviation from expected genotype distributions.

No statistically significant association was found between any individual SNP and clinical outcomes, including objective response, 3-month clinical benefit, or ≥G3 toxicity (**Supplementary Table 6**). For instance, rs28399433 showed a non-significant trend toward deviation from HWE (χ² = 2.98, *p* = 0.084), but did not correlate with objective response *(p* = 1.000), 3-month clinical benefit (*p* = 0.393), or ≥G3 toxicity (*p* = 0.707). Similarly, rs8192720 showed a borderline HWE deviation (χ² = 3.68, *p* = 0.055), but clinical correlations were non-significant.

## Discussion

Our prospective study provides safety and pharmacokinetic data on S-1 combined with cisplatin in patients with advanced cancers and varying degrees of hepatic dysfunction, including those with severe dysfunction. These patients are typically excluded from clinical trials and have limited treatment options. The safety profile observed across the four cohorts, together with preliminary pharmacokinetic findings and evidence of clinical activity in selected patients with severe hepatic dysfunction, suggests that this regimen may be feasible in selected patients and warrants further prospective evaluation.

5-FU pharmacokinetic analyses showed no significant differences between patients with normal and milder degrees of hepatic dysfunction; however, those with severe hepatic dysfunction (Cohort 4) exhibited lower peak concentrations (C_max_), longer time to reach maximum concentration (T_max_), and a non-significant trend towards lower 5-FU AUC at day 15 compared to patients with normal hepatic function (Cohort 1). These findings indicate altered 5-FU pharmacokinetic in patients with severe hepatic dysfunction. The lower C_max_ and prolonged T_max_ may be consistent with altered conversion of tegafur to 5-FU, but this mechanism was not directly evaluated in our study. Other factors affecting oral drug absorption, hepatic metabolism, or drug clearance may also contribute to the observed pharmacokinetic differences. Further studies incorporating direct measurement of tegafur and its metabolites, together with pharmacogenomic and pharmacokinetic analyses, would be required to elucidate the underlying mechanisms. Despite altered pharmacokinetics in severe hepatic dysfunction, overall 5-FU exposure remained comparable, and no significant association with toxicity or efficacy was observed. This is clinically meaningful, as it indicates that patients with severe hepatic dysfunction may receive effective therapy without experiencing excessive toxicity. Reassuringly, grade 3 or higher toxicities in patients with severe hepatic dysfunction were comparable to patients with less severe hepatic dysfunction and did not result in treatment-related deaths, underscoring the manageable safety profile of this regimen in patients with severe hepatic dysfunction.

In terms of efficacy, the ORR and 3M-CBR in Cohort 4 were noteworthy, with 22.2% of patients achieving a partial response and 40.7% deriving 3-month clinical benefit. The median PFS was also encouraging, with 22.2% of these patients maintaining PFS for six months or more. These findings indicate that objective responses and disease control were observed in a subset of patients despite severe hepatic dysfunction. However, these outcomes were secondary endpoints in this single-arm study with a small and heterogenous population, and the study was neither designed nor powered to establish treatment efficacy or to compare efficacy between hepatic-function cohorts. In addition, differences in tumor biology, prior treatment exposure, disease burden, and the underlying causes of hepatic dysfunction may have contributed to the observed outcomes. Accordingly, the efficacy findings should be regarded as preliminary and hypothesis-generating.

The etiology of hepatic dysfunction is an important consideration when interpreting studies of systemic therapy in patients with advanced cancer. Hepatic biochemical abnormalities may result from malignant hepatic involvement, biliary obstruction, infection, treatment-related injury, or underlying chronic liver disease, and these mechanisms may have different implications for treatment tolerance and drug pharmacokinetics. In the present study, active infection, including active hepatitis B or C infection and cholangitis, was excluded. Patients with malignant biliary obstruction were required to undergo biliary drainage before study entry, with repeat liver function testing performed after drainage before cohort assignment. Thus, potentially reversible infectious or obstructive causes were addressed where possible before baseline hepatic dysfunction was classified. Nevertheless, the study was not designed to prospectively distinguish the contribution of individual causes of hepatic dysfunction, particularly malignant hepatic infiltration or underlying chronic liver disease, and the small sample size precluded meaningful etiologic subgroup analyses. Accordingly, the observed safety, pharmacokinetic, and efficacy findings should not be attributed to any specific mechanism or cause of hepatic dysfunction.

In our study, we also investigated the association of CYP2A6 genetic polymorphisms with treatment response and toxicity in cancer patients receiving cisplatin plus S-1. Among the several variants with known or predicted functional impact on CYP2A6 identified, the prevalence of these variants in our study population was generally consistent with published data, with higher frequencies of the rs28399433 and rs5031016 alleles in East Asian populations compared to Western cohorts (15, 17). No statistically significant associations were observed between individual SNPs and clinical outcomes. However, these analyses were exploratory and hypothesis-generating, and should be interpreted cautiously given the modest sample size, incomplete availability of pharmacokinetic samples, and low frequency of several variants. The study was therefore not adequately powered to exclude potentially clinically relevant genotype–outcome associations. The absence of statistically significant findings should not be interpreted as definitive evidence of a lack of association. Nonetheless, our findings align with previous reports suggesting a limited role of CYP2A6 polymorphisms in modulating clinical outcomes with S-1 therapy, particularly in combination regimens where the pharmacokinetic complexity increases (18, 19). Furthermore, since cisplatin’s cytotoxic mechanism is not dependent on CYP2A6-mediated metabolism, any potential effect of CYP2A6 variants on treatment response may be attenuated. Inter-individual variability in S-1 metabolism – influenced by hepatic function, renal clearance, and polymorphisms in other metabolic enzymes – may also contribute to the lack of correlation observed. Future pharmacogenomic studies with larger cohorts, more comprehensive multi-gene panels, and integration of pharmacokinetic data may clarify the clinical relevance of CYP2A6 and other polymorphisms in patients receiving S-1–based therapy.

Treatment resistance represents another important consideration in interpreting the clinical outcomes observed in this study. No tumor-based molecular analyses of treatment resistance were prospectively performed, and therefore we cannot determine whether disease progression or limited clinical benefit in individual patients was attributable to specific resistance mechanisms. For fluoropyrimidine-based therapy, potential mechanisms include altered activation or catabolism of 5-FU, changes in thymidylate synthase and other enzymes involved in fluoropyrimidine metabolism, altered downstream nucleotide metabolism, and changes in cellular survival pathways (20–22). Drug transport mechanisms, including altered expression of ATP-binding cassette (ABC) transporters, have also been implicated in multidrug resistance, although their contribution to fluoropyrimidine resistance is complex and may vary according to the specific drug and tumor context (23). Resistance to cisplatin is similarly multifactorial and may involve altered drug transport, enhanced DNA damage repair, impaired apoptotic signaling, and changes in multiple cellular signaling pathways (24, 25). Thus, the clinical progression observed in some patients in our study cannot be mechanistically attributed to any specific resistance pathway. Future translational studies incorporating tumor genomic or transcriptomic profiling, together with functional assessment of fluoropyrimidine and platinum sensitivity, may help identify biomarkers associated with response or resistance to S-1 plus cisplatin.

This study has several important limitations. First, this was a single-arm study without a comparator arm and was not designed to determine comparative efficacy across hepatic-function cohorts. The secondary efficacy endpoints should therefore be regarded as exploratory and descriptive rather than evidence of treatment efficacy. Second, the study population was heterogeneous with respect to tumor type, prior treatment exposure, disease burden, and the clinical context underlying hepatic dysfunction. Tumor biology, sensitivity to S-1 and cisplatin, patterns of liver involvement, and natural history differ substantially between malignancies, limiting the interpretation of efficacy outcomes across tumor types. Breast cancer accounted for 57.4% of the study population and was overrepresented in Cohort 4; therefore, the observed efficacy outcomes in this subgroup should be considered exploratory and hypothesis-generating rather than indicative of tumor-specific efficacy. The eligibility criterion of tumors being “refractory to standard therapy or [having] no available therapies” was broad and did not define a standardized algorithm for determining whether all subsequent-line treatment options had been exhausted. Consequently, prior treatment exposure varied substantially across the study population, including patients with no prior systemic treatment and others who had received multiple previous lines. In some cases, enrolment reflected the clinical judgment of the treating physician in the context of the treatment landscape at the time, severe hepatic dysfunction limiting conventional treatment options, or individual patient factors. Furthermore, although active infection, including hepatitis B or C infection and cholangitis, was excluded and patients with malignant biliary obstruction underwent drainage before cohort assignment where applicable, the study was not designed to prospectively classify hepatic dysfunction according to etiology. The NCI-ODWG classification describes the severity of hepatic dysfunction but does not distinguish the contributions of malignant hepatic involvement, biliary obstruction, underlying chronic liver disease, prior treatment-related injury, or other causes of hepatic biochemical abnormalities. The small sample size precluded meaningful etiologic subgroup analyses. Third, this was a single-centre study conducted in Singapore, with 67.2% of patients identifying as Chinese. The predominantly Asian composition of the cohort, together with differences in pharmacogenomic profiles and treatment practices across populations, may limit the generalizability of the findings to other ethnic groups and healthcare settings. Although the pharmacokinetic findings provide prospective information on 5-FU exposure in patients with hepatic dysfunction, confirmation in larger and more ethnically diverse populations is required. Fourth, a substantial proportion of patients with moderate and severe hepatic dysfunction experienced rapid clinical deterioration, with 16.7% and 33.3% of patients in Cohorts 3 and 4, respectively, unable to complete even one cycle of study treatment. This reflects the challenges of conducting clinical trials in patients with advanced cancer and hepatic dysfunction, who often have poor prognosis and rapidly changing clinical status. Such early deterioration may have influenced safety, pharmacokinetic, and efficacy assessments. Finally, the study was closed after seven years because of slow accrual, and the eventual number of patients with available pharmacokinetic data at both predefined time points was below the original target sample size. Similarly, only 13 patients in Cohort 3 were evaluable for toxicity, short of the planned 15 patients. Incomplete pharmacokinetic sampling was partly attributable to rapid disease progression and clinical deterioration during early treatment cycles. These factors reduced the statistical power of exploratory analyses, particularly those examining associations between CYP2A6 polymorphisms, pharmacokinetic parameters, toxicity, and clinical outcomes. Accordingly, the pharmacogenomic analyses and pharmacokinetic–outcome correlations should be regarded as exploratory and hypothesis-generating rather than definitive.

These limitations are particularly relevant to interpretation of the secondary efficacy endpoints. The primary contribution of this study lies in its prospective evaluation of safety and 5-FU pharmacokinetics in patients with hepatic dysfunction, a population that is frequently excluded from clinical trials. Despite these limitations, prospective studies in patients with hepatic dysfunction remain challenging because such patients are frequently excluded from conventional clinical trials, and the present study provides descriptive safety and pharmacokinetic data in a population for whom evidence to guide treatment decisions remains limited.

In conclusion, the combination of S-1 and cisplatin was feasible in this prospective study of patients with varying degrees of hepatic dysfunction, including patients with severe hepatic dysfunction. Although more than one-third of patients with severe hepatic dysfunction experienced rapid disease progression and were unable to complete the first treatment cycle, clinically meaningful disease control and objective responses were observed among evaluable patients. These findings provide preliminary prospective evidence that cisplatin combined with S-1 may be a feasible treatment approach for selected patients with severe hepatic dysfunction who have limited therapeutic options. Given the single-arm design, modest sample size, heterogeneous tumor population, predominantly Asian study population and incomplete pharmacokinetic sampling, these findings should be interpreted cautiously and require confirmation in larger prospective studies.
