# Supplementary material for: Prospective evaluation of combination oral S-1 and intravenous cisplatin in cancer patients with severe hepatic dysfunction – a feasibility, safety, and pharmacokinetic study
Source: Front Oncol. 2026 Oct 16;16:1948986. doi: 10.3389/fonc.2026.1948986 (PMC13719379; doi:10.3389/fonc.2026.1948986)
Supplement: Supplementary file 1 [file DataSheet1.docx]

**Supplementary Table 1: Stratification of patients by hepatic dysfunction in accordance to the NCI-ODWG criteria**

| **Treatment Cohort** | **Degree of hepatic dysfunction** | **Total bilirubin level (pre-dose)** | **ALT/AST level (pre-dose)** |
| --- | --- | --- | --- |
| **Cohort 1** | Normal hepatic function  (control group) | ≤ULN | ALT and AST ≤ULN |
| **Cohort 2** | Mild hepatic dysfunction | ≤ULN | ALT and/or AST >ULN with both ALT and AST ≤ 5x ULN |
|  |  | >1.0-1.5x ULN | ALT and AST ≤ 5x ULN |
| **Cohort 3** | Moderate hepatic dysfunction | >1.5-3.0 x ULN | ALT and AST ≤ 5x ULN |
| **Cohort 4** | Severe hepatic dysfunction | >3.0-10.0 x ULN and/or | ALT and AST >5x and ≤ 20x ULN |

ALT: Alanine transaminase; AST: Aspartate transaminase; ULN: upper limit normal

**Supplementary Table 2: Patients by NCI-ODWG Hepatic Dysfunction Cohort and corresponding ALBI (Albumin-Bilirubin) Grade**

|  | | **Cohort** (N) | | | |
| --- | --- | --- | --- | --- | --- |
|  |  | **1**  (11) | **2**  (10) | **3**  (13) | **4**  (27) |
| **ALBI Grade**  N (%) | 1 | **4 (36%)** | **6 (60%)** | **0** | **0** |
|  | 2 | **7 (64%)** | **4 (40%)** | **12 (92%)** | **14 (52%)** |
|  | 3 | **0** | **0** | **1 (8%)** | **13 (48%)** |

**Supplementary Table 3: Exploratory Analysis Evaluating Correlation between 5-Fluorouracil Pharmacokinetic Parameters and Treatment Efficacy by Clinical Benefit (PR/SD for ≥3 months) Versus No Clinical Benefit, with Univariate and Multivariate Analysis**

|  | Clinical Benefit  (N = 20 for D8;  22 for D15) | No Clinical Benefit  (N = 18 for D8;  14 for D15) | Univariate Logistic Regression | Multivariate Logistic Regression |
| --- | --- | --- | --- | --- |
| D8 T_max_ – Mean (hours) | 2.00 | 2.94 | 0.025 | 0.150 |
| D8 C_max_ – Mean (ng/mL) | 192.67 | 149.14 | 0.051 | 0.478 |
| D8 AUC – Mean (h*ng/mL) | 672.32 | 598.14 | 0.279 |  |
| D15 T_max_ – Mean (hours) | 2.05 | 2.39 | 0.392 |  |
| D15 C_max_ – Mean (ng/mL) | 173.18 | 163.64 | 0.682 |  |
| D15 AUC – Mean (h*ng/mL) | 667.51 | 622.06 | 0.630 |  |

**Supplementary Table 4: Exploratory Analysis Evaluating Correlation between 5-Fluorouracil Pharmacokinetic Parameters and Treatment Toxicity, with Univariate and Multivariate Analysis**

|  | Patients with ≥G3 Toxicities  (N = 20 for D8;  17 for D15) | Patients with no ≥G3 Toxicities  (N = 21 for D8;  20 for D15) | Univariate Logistic Regression | Multivariate Logistic Regression |
| --- | --- | --- | --- | --- |
| D8 T_max_ – Mean (hours) | 2.050 | 2.810 | 0.055 | 0.259 |
| D8 C_max_ – Mean (ng/mL) | 184.970 | 167.833 | 0.425 |  |
| D8 AUC – Mean (h*ng/mL) | 723.454 | 586.137 | 0.059 | 0.298 |
| D15 T_max_ – Mean (hours) | 2.412 | 1.975 | 0.263 |  |
| D15 C_max_ – Mean (ng/mL) | 171.635 | 173.905 | 0.922 |  |
| D15 AUC – Mean (h*ng/mL) | 706.586 | 623.646 | 0.377 |  |

**Supplementary Table 5: Univariate and Multivariate Analyses of Cohort 4 patients by Objective Response**

|  | PR  (N = 6) | SD / PD  (N = 17) | Univariate Logistic Regression | Multivariate Logistic Regression |
| --- | --- | --- | --- | --- |
| Gender   - Male - Female | 1  5 | 6  11 | 0.406 |  |
| Age – Mean (years) | 57.7 | 56.7 | 0.857 |  |
| Histology   - Breast - Others | 5  1 | 9  8 | 0.213 |  |
| Prior lines of treatment | 2 | 3 | 0.772 |  |
| Total Bilirubin – Mean (x ULN) | 1.9 | 3.7 | **0.051** | 0.998 |
| AST – Mean (x ULN) | 7.0 | 6.6 | 0.841 |  |
| ALT – Mean (x ULN) | 5.5 | 2.2 | **0.026** | 0.991 |
| Albumin – Mean (g/L) | 33 | 32 | 0.429 |  |
| BMI – Mean (kg/m^2^ | 20.1 | 24.4 | 0.104 |  |
| D8 T_max_ – Mean (hours) | 1.75 | 3.00 | 0.138 |  |
| D8 C_max_ – Mean (ng/mL) | 213.50 | 126.68 | **0.056** | 0.993 |
| D8 AUC – Mean (h*ng/mL) | 733.15 | 519.96 | **0.094** | 0.993 |
| D15 T_max_ – Mean (hours) | 1.67 | 3.71 | 0.998 |  |
| D15 C_max_ – Mean (ng/mL) | 184.00 | 115.09 | 0.107 |  |
| D15 AUC – Mean (h*ng/mL) | 732.61 | 465.16 | 0.258 |  |

**Supplementary Table 6: CYP2A6 SNP Analysis and Correlation with Clinical Outcomes and Toxicity**

| **SNP ID** | **Change** | **Functional impact on CYP2A6** | **No. (%) of patients with SNP (out of 58)** | **Genotypes (observed)** | **HWE Testing** | | ***p*-value (χ² or Fisher’s)** | | |
| --- | --- | --- | --- | --- | --- | --- | --- | --- | --- |
|  |  |  |  |  | **χ² (or Fisher’s)** | ***p*-value** | **Objective Response** | **3-Month Clinical Benefit** | **≥G3 toxicity** |
| rs28399433 | A>C | ↓ mRNA expression | 21^a^ (36.2) | AA: 36  AC: 21 | 2.98 | 0.084 | 1.000 | 0.393 | 0.707 |
| rs8192720 | G>A | ↑ mRNA expression | 11 (19.0) | GG: 47  AG: 9  AA: 2 | 3.68 | 0.055 | 0.170 | 0.217 | 0.316 |
| rs28399440 | A>G | ↓ enzyme activity | 1^b^ (1.7) | AA: 56  AG: 1 | - | ~1.0 | 1.000 | 1.000 | 1.000 |
| rs5031016 | A>G | ↓ enzyme activity | 7^c^ (12.1) | AA: 50  AG: 7 | 0.24 | 0.62 | 0.582 | 1.000 | 0.706 |
| rs28399468 | C>A | Inactive enzyme | 2^d^ (3.4) | CC: 55  AC: 2 | - | ~1.0 | 1.000 | 1.000 | 0.491 |

^a-d^one patient with unavailable result

HWE: Hardy-Weinberg Equilibrium

**Supplementary Figure 1: Swimmer’s plot illustrating the treatment timeline for patients in Cohort 4 who were evaluable for response**

**Supplementary Figure 2: Trends of Hepatic Function Parameters (A) Total Bilirubin (B) Aspartate Transaminase (C) Alanine Transaminase at Baseline and After One and Two Cycles of Study Treatment**
